# Efficacy of rituximab therapy in children with nephrotic syndrome: a 10-year experience from an Iranian pediatric hospital

**DOI:** 10.1186/s12887-022-03109-4

**Published:** 2022-01-12

**Authors:** Behnaz Bazargani, Zahra Noparast, Leila Khedmat, Daryoosh Fahimi, Seyed Taher Esfahani, Mastaneh Moghtaderi, Arash Abbasi, Azadeh Afshin, Sayed Yousef Mojtahedi

**Affiliations:** 1 Pediatric Chronic Kidney Disease Research Center, The Children’s Hospital Medical Center, Tehran University of Medical Sciences, Tehran, Iran; 2 Department of Pediatrics, Division of Nephrology, Children’s Hospital Medical Center, Tehran University of Medical Sciences, Tehran, Iran; 3 Department of Pediatric Nephrology, Bahrami Children Hospital, Tehran University of Medical Sciences, Tehran, Iran; 4 Health Management Research Center, Baqiyatallah University of Medical Sciences, Tehran, Iran

**Keywords:** Rituximab, Nephrotic syndrome, Pediatrics, Steroid dependent nephrotic syndrome, Steroid resistant nephrotic syndrome

## Abstract

**Background:**

There are controversy results in the optimal management of children with steroid-dependent and steroid-resistant nephrotic syndrome (SDNS, SRNS). This study aimed to determine the efficacy and safety of rituximab (RTX) in these pediatric patients.

**Methods:**

Medical records of 1–18-year-old Iranian children with SDNS (*n* = 26) and SRNS (*n* = 22) with a follow-up for at least 24 months were included from 2009 to 2019. The short- and long-term responses to RTX were respectively evaluated to determine the random protein-to-creatinine ratio after 6 and 24 months and classified as complete (CR) and partial (PR) remission or no response.

**Results:**

Male patients (*n* = 26) were slightly predominate. The median age of patients at the time of RTX therapy was 8.6 ± 4.01 years. At the end of the 6-month follow-up, CR and PR occurred in 23 (47.9%) and 12 (25%) patients, respectively. Of 23 patients with CR, 18 (69.2%) and 5(22.7%) had SDNS and SRNS, respectively (*p* < 0.005). However, only 18 (37.5%) of patients after 24 months had been in CR. No significant difference in the CR rate was found between the two groups. RTX was more effective when administered during the proteinuria-free period (*p* = 0.001).

**Conclusion:**

In the short term, RTX significantly was efficient in inducing complete or PR in SDNS and SRNS patients. However, the favorable response rate in a long-term follow-up was insignificantly lower between the two groups.

## Introduction

Nephrotic syndrome (NS) is defined as heavy or nephrotic proteinuria (urine protein > 40 mg/m^2^/h or random urine protein-to-creatinine ratio (urine Pr/Cr) > 2 mg/mg), hypoalbuminemia (< 25 g/L), hypercholesterolemia (> 250 mg/dL), and generalized edema [1]. The idiopathic form of this disorder (INS) constitutes 90% of childhood NS with the globally estimated prevalence of 2.0–16.9 per 100,000 children [2, 3]. The prevalence rate of INS differs in different parts of the world. Sharples et al. reported that this syndrome in Asian children is six times more than Caucasian ones [4]. Changing the glomerular permeability in INS causes an alteration in the plasma protein sieving coefficient [5, 6]. Immunoglobulin deposits at the mesangium are present in one-third of patients with minimal change disease (MCD) [7, 8]. Genetic predisposition, circulatory factors, and infections could contribute to the development of INS in an immunologically susceptible patient [9, 10]. An international study of kidney disease in children (ISKDC) defined the steroid-dependent nephrotic syndrome (SDNS) as the two consecutive relapses during the tapering or within the 14 days following the termination of the steroid therapy [11, 12]. The alternative therapeutic agents used for SDNS were cyclophosphamide (CP), mycophenolate mofetil (MMF), and calcineurin inhibitors (CNIs) [13]. Steroid resistant nephrotic syndrome (SRNS) is defined as persistent heavy proteinuria 4–6 weeks after an oral prednisolone therapy [12]. 10–20% of INS patients may also be resistant to CNIs and alkylating agents [14, 15] and thus are at high risk of end-stage renal disease [16].

Rituximab (RTX) is a chimeric anti-CD20 monoclonal antibody, which inhibits CD20-mediated B-cell proliferation and differentiation, resulting in depletion of peripheral blood B lymphocytes [17–19]. Several studies showed promising effects of RTX in achieving complete remission (CR) or partial remission (PR) in SDNS and SRNS patients by showing either discontinuation or reduction of steroid and/or immunosuppressive therapy [17–21]. Recently, RTX with different effectiveness rates has been using for the treatment of patients with SDNS and SRNS [22]. RTX can be directly bonded to the acid sphingomyelinase-like phosphodiesterase 3b on the podocytes, leading to the stabilized podocyte function and structure to prevent future recurrence [23]. Moreover, this chimeric monoclonal antibody regulates the cytoskeleton and regulatory elements of CD20 positive B cells. The regulatory T cell impairment and the remission induction by RTX have been indicated in previous studies [24]. RTX increased the number and function of regulatory T cells [25]. On the other hand, RTX reduces the proliferation of B cells through their apoptosis induction. Accordingly, the function of B cells and consequently their interaction with T cells will be suppressed to prevent the future recurrence of INS [24, 25]. Colucci et al. reported that the delayed reconstruction of B cells is associated with a lower risk of relapse, independent of the administered immunosuppressive therapy [26].

Although clinical studies related to the effect of RTX in improving the therapy and outcomes of children with SDNS have been recently increased [27–29], there is little evidence on the long-term results of concomitant treatments, renal pathology (RP), and clinical outcomes in the early and late follow-up period among Iranian children with SDNS and SRNS within a wide age range. Therefore, the present 10-year study was assessed to investigate the effectiveness of RTX on Iranian pediatric patients with SDNS and SRNS in terms of the proteinuria degree.

## Material and methods

### Study design and participants

This study was a retrospective chart review conducted at Children's Medical Center (Tehran, Iran) from March 2009 to February 2019. Forty-eight pediatric patients with SDNS (*n* = 26) and SRNS (*n* = 22) within the age range of 1 to 18 years were participated according to the census sampling method. After mentioning objectives and used methodologies for the present research, both the verbal and written informed consents from all the parents were obtained through phone contacts and face-to-face interviews. This study was performed following the Declaration of Helsinki and approved by the Human Ethics Committee of the Tehran University of Medical Sciences (TUMS).

### Inclusion and exclusion criteria

Patients who did not respond well to drugs (such as steroids (e.g., prednisolone) or immunosuppressive agents (e.g., CNIs, MMF, and CP)) or develop complications and side effects were entered in the study. However, patients who did not receive four doses of RTX were also excluded from the study. Also, patients with secondary forms of NS, glomerular filtration rate < 60 mL/min/1.73 m^2^, and with less than 2 years of follow-up were excluded.

### NS diagnosis and RTX treatment

All patients had previously undergone kidney biopsy. Although there was no requirement for biopsy of patients with SDNS, these patients with disease recurrence and the onset of complications underwent a biopsy before RTX administration as the final stage of treatment. The diagnosis of the underlying renal histopathology had been made by light, immunofluorescence, and electron microscopic evaluation. Before the RTX treatment, the urine protein to creatinine ratio (Pr/Cr, mg/dL) was randomly measured and patients were subsequently categorized into physiologic (urine Pr/Cr < 0.2), sub-nephrotic (0.2 < urine Pr/Cr < 2.0), and nephrotic proteinuria (urine Pr/Cr > 2.0). Four doses of RTX were given (375 mg/m^2^/dose) 1 week apart and patients were followed for random urine Pr/Cr, at one-month intervals for 6 months and thereafter every 3 months for a minimum of 2 years. Early and late response (respectively at 6 months and 2 years of follow up) to RTX were defined as CR (urine Pr/Cr ≤ 0.2) or PR (0.2 < urine Pr/Cr ≤ 2.0 and no edema), and no response (urine Pr/Cr > 2.0). Prophylaxis with cotrimoxazole as a low-risk approach was also used to prevent pneumocystis pneumonia (PCP) in high-risk patients.

### Data analysis

Results were analyzed using SPSS 20.0 software (SPSS Inc., Chicago, IL, USA). The student’s *t*-test was used to compare between groups and represented as a 95% confidence interval. Differences with a *p*-value lower than 0.05 were regarded as significant.

## Results

During the study period, 51 cases with INS were candidates for RTX treatment. Among these patients, 48 subjects (26 male and 22 female) received four doses of RTX. Characteristics of patients and their response to RTX therapy are shown in Table 1. The mean age of patients was 9.17 ± 2.30 years, while they were followed for 6 to 118 months (38.45 ± 6.63). There was no significant difference between the two groups according to age and gender. Before the initiation of therapy, patients with SRNS showed more severe degrees of proteinuria than the SDNS group (*p* = 0.049). Although SDNS patients had a more favorable response than SRNS in the sixth month (*p* = 0.005), there was no significant difference between them after 24 months (*p* = 0.101). The response rate of SRNS patients was not changed during this time interval. RTX was more effective in inducing a long-term remission when administered during a proteinuria-free period (*p* = 0.001). The response time following the initial dose was 1.8 ± 2.82 and 1.27 ± 1.61 months in SRNS and SDNS groups, respectively (*p* = 0.372). Among the female population, the CR rate was significantly higher in the SDNS group compared to SRNS one (*p* = 0.001). However, this index was not significant among males (*p* = 0.417). Complete (55.6%) and partial (83.3%) remission rates were more observed in patients with diffuse mesangial proliferation (DMP) pathology (*p* = 0.003).

**Table 1:** Baseline characteristics of children patients with SDNS and SRNS

Characteristics	N_**total**_	SDNS (n)	SRNS (n)	***P*** value
**Gender**	48	26	22	0.529
Male	26 (54.2%)	13 (50.0%)	13 (59.1%)	
Female	22 (45.8%)	13 (50.0%)	9 (40.9%)	
**Age at onset of treatment (yr)**	48 (9.17 ± 2.30)	26 (9.42 ± 0.21)	22 (7.64 ± 0.32)	0.126
**Proteinuria before RTX treatment**				0.049
Nephrotic	24 (50.0%)	9 (34.6%)	15 (68.2%)	
Sub-nephrotic	18 (37.5%)	12 (46.2%)	6 (27.3%)	
Physiologic	6 (12.5%)	5 (19.2%)	1 (4.5%)	
**Renal pathology**^a^				0.016
MCNS	5 (10.4%)	4 (15.4%)	1 (4.5%)	
FSGS	15 (31.2%)	4 (15.4%)	11 (50.0%)	
DMP	27 (56.2%)	18 (69.2%)	9 (41.0%)	
MGN	1 (2.1%)	0 (0.0%)	1 (4.5%)	
**Concomitant treatments**^b^				0.302
Steroid	4 (8.3%)	1 (3.8%)	3 (13.7%)	
CNI	11 (22.9%)	6 (23.1%)	5 (22.7%)	
MMF	19 (39.6%)	13 (50.0%)	6 (27.3%)	
CNI plus MMF	14 (29.2%)	6 (23.1%)	8 (36.3%)	
**Early outcome (6 months after RTX)**				0.005
Complete remission	23 (47.9%)	18 (69.2%)	5 (22.7%)	
Partial remission	12 (25.0%)	3 (11.6%)	9 (41.0%)	
No response	13 (27.1%)	5 (19.2%)	8 (36.3%)	
**Outcome at last follow-up**				0.101
Complete remission	18 (37.5%)	13 (50.0%)	5 (22.7%)	
Partial remission	18 (37.5%)	9 (34.6%)	9 (41.0%)	
No response	12 (25.0%)	4 (15.4%)	8 (36.3%)	
**Existence of complication**				0.196
Yes	15 (31.2%)	10 (38.5%)	5 (22.7%)	
No	33 (68.8%)	16 (61.5%)	17 (77.3%)	
**Disease duration (month)**^c^	48 (54.54 ± 37.12)	26 (67.12 ± 39.95)	22 (39.68 ± 34.27)	0.015
**Therapeutic response onset (month)**^d^	48 (1.55 ± 1.86)	26 (1.27 ± 1.61)	22 (1.88 ± 2.82)	0.372
**The first relapse time (month)**	35 (3.88 ± 4.51)	21 (4.10 ± 5.47)	14 (3.57 ± 7.12)	0.807

^a^*MCNS* Minimal change nephrotic syndrome, *FSGS* Focal and segmental glomerulosclerosis, *DMP* Diffuse mesangial proliferation, *MGN* Membranous glomerulonephritis

^b^*CNI* Calcineurin inhibitor, *MMF* Mycophenolate mofetil

^c^From diagnosis to administration

^d^ After the first injection of RTX

The mean interval between the diagnosis of INS and the first RTX administration was determined to be 54 ± 39 months. The disease duration for patients with SDNS and SRNS was 67.12 and 39.68 months, respectively. Thus, SDNS patients had a longer disease duration than SRNS ones (*p* = 0.015). However, Table 1 shows that there was no significant difference in the therapeutic response onset between patients with SDNS (1.27 month) and SRNS (1.88 month). There was no significant correlation between this interval and the rate of remission (*p* = 0.438). The first relapse occurred after 4.1 ± 5.47 and 3.57 ± 7.12 months following the RTX therapy for patients with SDNS and SRNS, respectively (*p* = 0.807). Fifteen (31.2%) patients had side effects of RTX infusion including fever, rash, hypotension, and dyspnea, without any mortality. In general, there was no significant difference in the complication existence among pediatric patients adminitered with RTX (*p* = 0.196). The existence of complication was reported to be 38.5 and 22.7% for patients with SDNS and SRNS, respectively (Table 1). Only two patients with SDNS, allergic to cotrimoxazole prophylaxis, developed PCP. After the RTX treatment, patients were placed on maintenance therapy with a low dose of prednisolone alone in 4 (8.3%) or combination with CNI in 11 (22.9%), MMF in 19 (39.6%), and CNI plus MMF in 14 (29.2%).

**Fig. 1:**
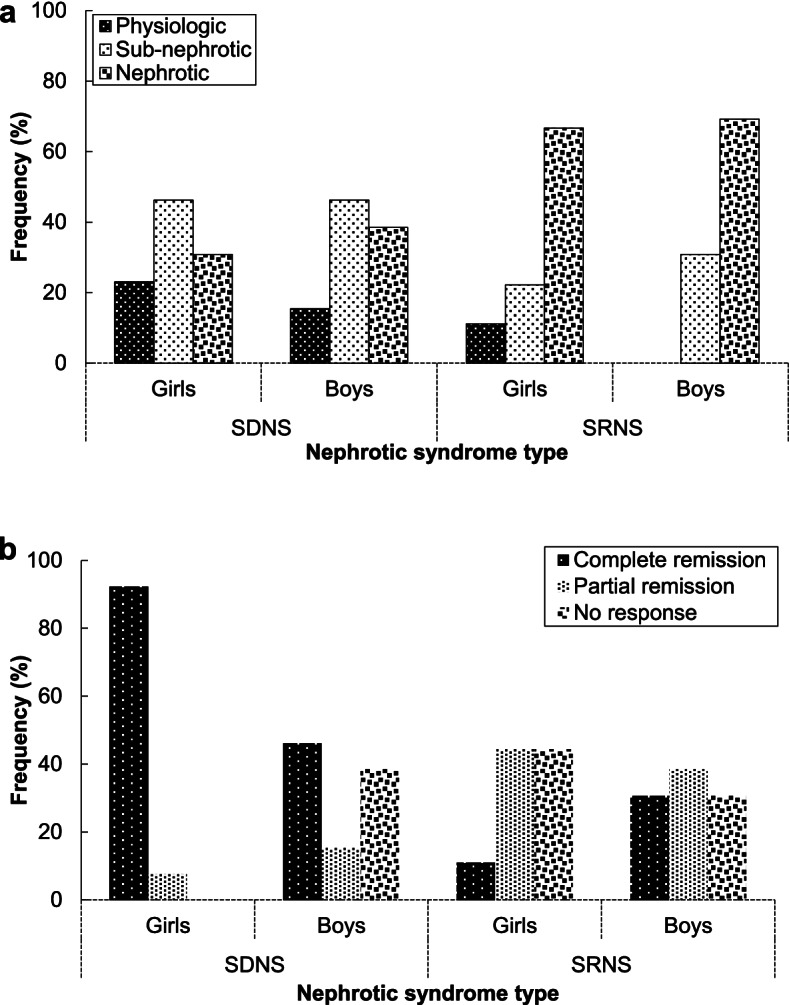
The relationship of NS type in both genders with (**a**) proteinuria before RTX treatment and (**b**) early outcome in pediatric patients

**Fig. 2:**
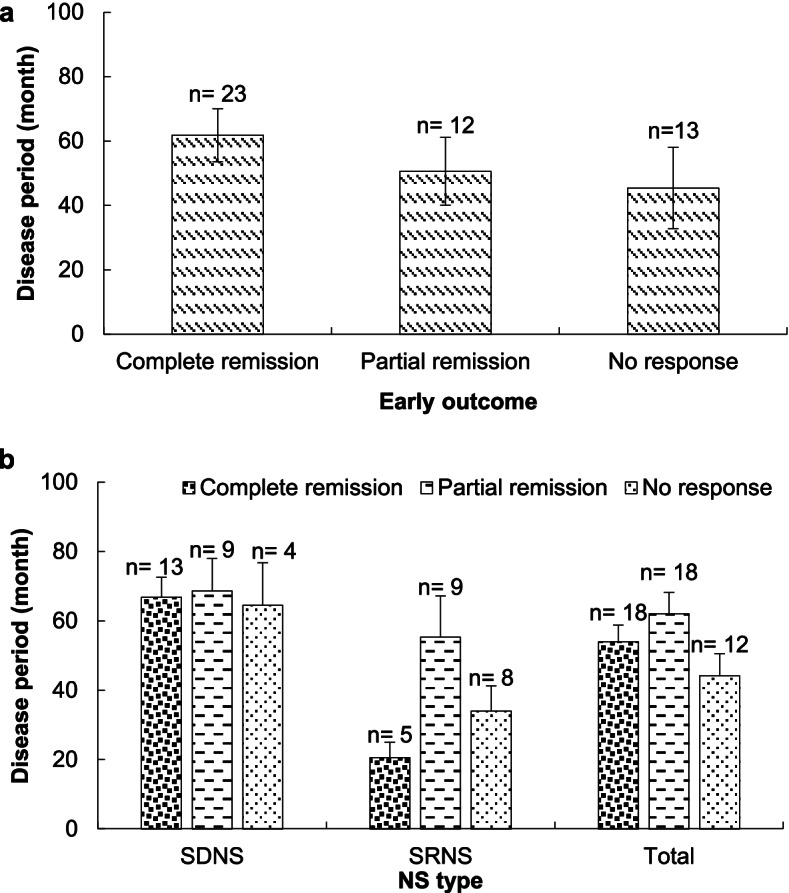
The mean comparison of disease duration based on early (**a**) and final (**b**) treatment outcomes of RTX in Iranian pediatric patients with NS

Figure 1 illustrates the association of NS type in both genders with proteinuria before RTX treatment and early outcome in pediatric patients. Most girls and boys with SDNS showed sub-nephrotic proteinuria, while the nephrotic type was the most common proteinuria among boys and girls with SDNS. Also, no physiologic proteinuria was observed among boys with SRNS (Fig. 1a). Not only a notable percentage of girls with SDNS (92.3%) were completely recovered, but there was no negative outcome among girls with SDNS. However, 38.5% of boys with SDNS did not respond to the RTX treatment. Among patients with SRNS, boys compared to girls after the RTX treatment were more recovered completely (Fig. 1b). Figure 2 compares the disease duration based on early and final treatment outcomes of RTX among patients with NS. As an early outcome of RTX administration, 47.9% of patients with NS showed a CR during 61.78 months (Fig. 2a). The final outcome of RTX treatment also shows that there was the same number of NS patients with complete or PR. Also, more SDNS patients than SRNS ones showed a CR during a longer disease period (Fig. 2b).

Table 2 represents the link between treatment outcome and proteinuria before RTX administration among patients with SDNS and SRNS. There was no significant association between proteinuria before RTX treatment and treatment outcome among patients with SDNS, while this relationship was significantly found among patients with SRNS (*p* = 0.005; Table 2). Totally, a considerable association was obtained between proteinuria before RTX administration and treatment outcome in patients with NS (*p* = 0.001; Table 2). The association between proteinuria before RTX treatment and RP was also shown in Table 2. There was no significant relationship between SDNS and RP. Nevertheless, SRNS (*p* = 0.047) and SDNS+SRNS (*p* = 0.003) were significantly correlated to the RP. DMP and FSGS were the most frequent RP among patients with SDNS and SRNS, respectively (Table 2). The most abundant RP type among the total population of patients with NS was DMP (56.2%), FSGS (31.2%), MCNS (10.4%), and MGN (2.1%), respectively (Table 2).

**Table 2:** The relationship between treatment outcome and proteinuria before RTX administration and renal pathology in pediatric patients with NS

NS type	Proteinuria before RTX treatment	Outcome			Total
		Complete remission	No response	Partial remission	
SDNS (*p* = 0.145)	Nephrotic	5 (38.5%)	0 (0.0%)	0 (0.0%)	5 (19.2%)
	Sub-nephrotic	4 (30.8%)	2 (50.0%)	6 (66.7%)	12 (46.2%)
	Physiologic	4 (30.8%)	2 (50.0%)	3 (33.3%)	9 (34.6%)
*Total*		13 (100%)	4 (100%)	9 (100%)	26 (100%)
SRNS (*p* = 0.005)	Nephrotic	1 (20.0%)	0 (0.0%)	0 (0.0%)	1 (4.5%)
	Sub-nephrotic	0 (0.0%)	0 (0.0%)	6 (66.7%)	6 (27.3%)
	Physiologic	4 (80.0%)	8 (100%)	3 (33.3%)	15 (68.2%)
*Total*		5 (100%)	8 (100%)	9 (100%)	22 (100%)
SDNS+SRNS (*p* = 0.001)	Nephrotic	6 (33.3%)	0 (0.0%)	0 (0.0%)	6 (12.5%)
	Sub-nephrotic	4 (22.2%)	2 (16.7%)	12 (66.7%)	18 (37.5%)
	Physiologic	8 (44.4%)	10 (83.3%)	6 (33.3%)	24 (50.0%)
*Total*		18 (100%)	12 (100%)	18 (100%)	48 (100%)
**NS type**	**Renal pathology**^a^	**Outcome**			**Total**
		**Complete remission**	**No response**	**Partial remission**	
SDNS (*p* = 0.113)	MCNS	3 (23.1%)	1 (25.0%)	0 (0.0%)	4 (15.4%)
	DMP	9 (69.2%)	1 (25.0%)	8 (88.9%)	18 (69.2%)
	FSGS	1 (7.7%)	2 (50.0%)	1 (11.1%)	4 (15.4%)
	MGN	0 (0.0%)	0 (0.0%)	0 (0.0%)	0 (0.0%)
*Total*		13 (100%)	4 (100%)	9 (100%)	26 (100%)
SRNS (*p* = 0.047)	MCNS	1 (20.0%)	0 (0.0%)	0 (0.0%)	1 (4.5%)
	DMP	1 (20.0%)	1 (12.5%)	7 (77.8%)	9 (40.9%)
	FSGS	3 (60.0%)	6 (75.0%)	2 (22.2%)	11 (50.0%)
	MGN	0 (0.0%)	1 (12.5%)	0 (0.0%)	1 (4.5%)
*Total*		5 (100%)	8 (100%)	9 (100%)	22 (100%)
SDNS+SRNS (*p* = 0.003)	MCNS	4 (22.2%)	1 (8.3%)	0 (0.0%)	5 (10.4%)
	DMP	10 (55.6%)	2 (16.7%)	15 (83.3%)	27 (56.2%)
	FSGS	4 (22.2%)	8 (66.7%)	3 (16.7%)	15 (31.2%)
	MGN	0 (0.0%)	1 (8.3%)	0 (0.0%)	1 (2.1%)
*Total*		18 (100%)	12 (100%)	18 (100%)	48 (100%)

^a^*MCNS* Minimal change nephrotic syndrome, *FSGS* Focal and segmental glomerulosclerosis, *DMP* Diffuse mesangial proliferation, *MGN* Membranous glomerulonephritis

## Discussion

This study showed the promising efficacy of RTX in SDNS and SRNS patients, especially in the former group. MCD was observed in most pediatric patients with INS. However, DMP and focal and segmental glomerulosclerosis (FSGS) were more common as more severe forms of SDNS and SRNS cases had been participated in the present study.

According to the study of Sinha et al., children with FSGS are at a higher risk of being non-responsive to RTX [30]. On the other hand, Magnasco et al. [31] found that there was no association between underlying histological abnormality and a response to the treatment. The current study indicated that the remission rate was significantly higher among patients with DMP. Previous studies also had shown that 10 to 20% of SDNS patients who responded to cyclosporine experienced frequent relapses [32, 33], while approximately 30% of children with SRNS after achieving CR had frequent, late-steroid, sensitive relapses [34]. In contrast, CNI administration was associated with multiple adverse effects, including glucose intolerance and chronic nephrotoxicity [35]. These findings indicate the importance of using alternative treatment approaches. Some studies recommended RTX for FSGS patients who are steroid- and CNIs-resistant [36].

In an international multicenter study, the efficacy of RTX to treat 28 patients with SDNS and 27 ones with SRNS was shown [17]. In contrast, Fernandez-Fresnedo et al. did not observe any beneficial effect in 75% of patients and fair effects in others [37]. Similarly, Magnasco et al. reported that RTX was not effective for patients with INS, who were resistant to steroids and CNIs [31]. Gulati et al. found a reduction in proteinuria and normalization of serum albumin in 80 and 44% of refractory SRNS patients after administering RTX at initial assessment and the end of 5-month follow-up, respectively [38]. In the present study, the response rate of the SDNS group was decreased in time, but it did not change in SRNS one. Based on these findings, RTX may be more commonly used to treat INS patients with an unfavorable response to conventional therapies who suffer from the protracted clinical course.

Maxted et al. by comparing single dose to four doses of RTX showed similar effects on inducing remission at 6-month follow-up. However, it was less effective at 12- and 24-month follow-up periods [39]. Hogan et al. indicated that the higher dose of RTX was associated with a lower risk of relapse [40]. Likewise, Kemper et al. explained that the time to the first relapse was significantly shorter for lower used doses [41]. Iijima et al. in a randomized placebo-controlled trial showed that four doses of 375 mg/m^2^ of RTX led to the relapse-free interval of 267-day compared to 101-day of placebo (*p* < 0.0001) [42]. In line with previous studies, the improved relapse-free interval has been reported by two meta-analyses [43, 44]. Moreover, RTX may be effective in a large subset of SDNS patients, especially when used in combination with other immunosuppressive drugs during a proteinuria-free period [19, 45]. In this study, RTX also was effective in patients when administered during a proteinuria-free period.

Sinha et al. reported that the remission duration significantly was shorter for patients with SRNS [30]. Nonetheless, Hogan et al. found that the risk of relapse following RTX administration was more in patients with higher levels of steroid dependency [40]. Hoseini et al. reported that the dependence on the steroid unlike age, sex, and underlying pathology could affect the response to RTX [46]. Kamei et al. indicated that the combination of RTX and methylprednisolone induced remission in refractory cases of SRNS [47]. In our study, the 6–month CR rate was more frequent in SDNS than SRNS, whereas the complete recovery rate was more frequent for SDNS than SRNS at the end of follow-up. The response rate of SRNS patients was not changed during this time interval. Thus, it was hypothesized that dependency is an important factor for response to RTX therapy.

Following the RTX administration, relapse episodes decreased by 62–95% [17, 38]. The PR and CR rates were achieved in 21.2–37.5% and 0–27.3% cases, respectively [22]. In this study, the complete and PR rate after 6-month was 47.9 and 25%, respectively. The corresponding data respectively was 37.5 and 37.5% at the end of follow-up time. The recovery rate was reported to be 83.3% of the cases [38]. Kimata et al. indicated that the 4 times use of RTX during a 3-month period resulted in a long-term recovery without any adverse effects [48]. Basu et al. pointed out the recovery of 33% of patients with the combined administration of RTX and MMF compared to the single-use of RTX. In our study, the recovery occurred in 75% of the patients (37.5% with complete recovery and 37.5% with partial recovery). CNI was administered for 22.9% cases, MMF for 39.6%, CNI and MMF for 29.2%, and prednisolone alone for 8.3%. However, the recovery rate did not differ among drug regimens [49].

Lethal complications of RTX include death due to pulmonary fibrosis [50] and fulminant myocarditis [51]. In a multicentric study conducted by Guigonis et al. [19], transient adverse reactions were reported in 45% of infusions. Other complications were reversible cytokine shock, neutropenia without severe infection [51], anaphylaxis, and serious infections [17]. Nevertheless, none of the patients in this study experienced any serious adverse effects.

## Conclusion

This study evaluated the improving effect of RTX on Iranian children patients with SDNS and SRNS. Results showed that this monoclonal antibody in a short-term follow-up period could successfully act as a CR or PR for pediatric patients with SDNS and SRNS. There was no remarkable difference in the short-term effectiveness rate of RTX between the two groups. However, the low number of patients treated with RTX showed CR after a 24-month follow-up. Besides, the highest effectiveness of this targeted therapy occurred after its administration during the proteinuria-free period. In general, it seems that some combined treatments with low-dose RTX, such as adjunct immunosuppressive therapies, would be necessary to induce more effectiveness in the therapy of children with INS. Since this study was conducted in the presence of a small number of patients, future larger-scale clinical studies are recommended to assess the efficiency and safety of RTX in treating INS in pediatrics. Moreover, the genetic diagnostic tests of patients with SRNS were not performed due to some limitations in the hospital such as high cost and low accessibility. The implementation of genetic tests at least in children with SRNS is recommended for potential future investigations. These experiments with the recognition of specified mutations allow the prediction and further screening of renal and extra-renal comorbidities with a shorter diagnostic process [52]. The assessment of serious cancerous tumors (e.g., Wilms’ tumor or gonadoblastoma in patients with WT1 mutations) can be potentially possible through genetic diagnostic tests for the primary analysis and disease monitoring of certain extra-renal phenotypes [53]. Another benefit of using genetic tests is to prevent detrimental treatments such as the administration of common steroids or immunosuppressants or to help the better treatment patterns for the future [52, 54]. Furthermore, the genetic diagnostic approach not only contributes to identify rare mutations but also predicts the probability of post-transplant recurrence of focal segmental glomerulosclerosis [54, 55]. Accordingly, an accurate molecular diagnosis with identifying new SRNS genes and causative mutations may better identify the pathogenic pathways and effective treatment plans of SRNS.

## Data Availability

The datasets used and/or analyzed during the current study are available from the corresponding author on reasonable request.

## Abbreviations

CNI: Calcineurin inhibitor
CP: Cyclophosphamide
CR: Complete remission
DMP: Diffuse mesangial proliferation
FSGS: Focal and segmental glomerulosclerosis
(I)NS: (Idiopathic) nephrotic syndrome
MCD: Minimal change disease
MMF: Mycophenolate mofetil
PCP: *Pneumocystis* pneumonia
PR: Partial remission
RP: Renal pathology
RTX: Rituximab
SDNS: Steroid-dependent nephrotic syndrome
SRNS: Steroid-resistant nephrotic syndrome
